# *Avicennia marina* endogenous promoter AMGT1P33 enhances salt tolerance in Arabidopsis by regulating exogenous salt-tolerance genes

**DOI:** 10.3389/fpls.2025.1541465

**Published:** 2025-03-14

**Authors:** Yi Wang, Shuwen Jia, Xinze Xu, Jie Shen, Jian Zhang, Zefu Cai, Shiquan Chen

**Affiliations:** ^1^ Institute of Marine Ecology, Hainan Academy of Ocean and Fisheries Sciences, Haikou, China; ^2^ Key Laboratory for Coastal Marine Eco-Environment Process and Carbon Sink of Hainan Province, Yazhou Bay Innovation Institute, College of Ecology and Environment, Hainan Tropical Ocean University, Sanya, China; ^3^ Qukou Scientific Research Base, Institute of Marine Ecology, Hainan Academy of Ocean and Fisheries Sciences, Haikou, China; ^4^ The French Associates Institute for Agriculture and Biotechnology of Drylands, The Jacob Blaustein Institutes for Desert Research, Ben-Gurion University of the Negev, Sede Boqer, Israel

**Keywords:** *Avicennia marina*, AMGT1P33, endogenous promoter, salt-tolerance mechanism, functional verification, monocotyledons, dicotyledons

## Abstract

**Introduction:**

Mangroves form ecologically and economically important ecosystems and are a potential source of valuable genetic resources given their natural salt tolerance. However, the role that promoters play in their salt-tolerance mechanisms remains unclear.

**Methods:**

In this study, we identified the AMGT1P33 promoter in the genome of the mangrove tree species Avicennia marina using PromPredict and then verified its promoter function according to the transient expression of GUS. Subsequently, the characteristics of AMGT1P33 and its involvement in salt tolerance were investigated.

**Results:**

Analysis of the transcription range showed that AMGT1P33 regulates GUS expression in both dicotyledonous (Nicotiana tabacum, Pachyrhizus erosus, and Solanum tuberosum) and monocotyledonous (Agropyron cristatum, Cocos nucifera, and Thalassia hemprichii) plant species. According to quantitative real-time-PCR, the expression level of GUS in N. tabacum when regulated by AMGT1P33 was 5.97 times higher than that when regulated by the 35S promoter. Additionally, the regulation of AmBADH expression by AMGT1P33 in yeast and Arabidopsis significantly improved salt tolerance.

**Discussion:**

These findings suggest that endogenous salt-tolerance-related promoters play a key role in the salt-tolerance mechanism of A. marina. These findings can be extended to elucidate the salt-tolerance mechanisms in other plants and contribute to the development of new promoter tools and methods for transgenic engineering.

## 1 Introduction

Plants are sessile organisms subjected to various biotic and abiotic stresses, which collectively contribute to more than 50% of global crop losses (Kumar and Saddhe, 2018). Salinity is a major abiotic factor affecting plant growth and development (Kumar and Saddhe, 2018). Currently, approximately 60% of global land is affected by salt accumulation (Hafeez et al., 2021), posing a major threat to modern agriculture. Over 2% of all land and nearly 20% of irrigated agricultural land experience high salinity levels (Isayenkov, 2019), leading to huge economic losses that exceed 10 billion USD annually (Qadir et al., 2014).

Halophytes are plants that are capable of growth even under saline conditions (Hafeez et al., 2021). They comprise 1–2% of the world’s flora and are both monocotyledonous and dicotyledonous (Kumari et al., 2015). Halophytes play an important role in maintaining ecological balance and provide abundant genetic resources for sugar-based crops that are beneficial for enhancing human food security. As a type of obligate halophyte, mangroves form unique tropical/subtropical ecosystems that grow in tidal wetlands (Woodroffe et al., 2016). Their living environment is characterized by extreme, complex, and variable characteristics such as high salinity, intense ultraviolet radiation, drought, and hypoxia (Kathiresan and Bingham, 2001; Su et al., 2019; Fei et al., 2021; Yu et al., 2021). Mangroves grow well under both extreme salinity stress and freshwater conditions and may be the only plant system that is broadly adapted to such extreme conditions (El-Atawy et al., 2021). As well as exhibiting high productivity, recovery, decomposition, and resistance rates (Liu and Wang, 2020), mangrove ecosystems provide key ecosystem services (Peng et al., 2020a, 2020b), such as providing food and shelter for marine life (Pootakham et al., 2022a), protection against natural disasters such as hurricanes and tsunamis (Pootakham et al., 2022b), shoreline protection (Schmitt and Duke, 2015), and remediation of polluted environments (Theuerkauff et al., 2020).

As naturally salt-tolerant plant species that have evolutionarily adapted to intertidal coastal ecosystems, mangroves possess abundant and valuable genetic resources (Saddhe and Kumar, 2019; Kathiresan, 2020). However, previous research on mangroves has mostly focused on their ecological functions, with relatively few molecular analyses (Zhang et al., 2021a). Despite successive reports on salt-tolerance genes and their corresponding pathways in mangroves, mangroves remain an underutilized source of salt-tolerance genes (Augustine et al., 2023), and their salt-tolerance mechanism remains underexplored (Zhang et al., 2021a). Thus, understanding the salt-tolerance mechanisms and development of salt-tolerance genes in mangroves may indicate ways to improve salt tolerance in other plants, develop saline–alkali land, and ensure human food security. Research on the mechanisms of salt tolerance in plants has focused on salt-tolerance genes (single or polygenic) (Pan et al., 2021; Zheng et al., 2021), transcription factors (Krishnamurthy et al., 2019; Pradhan et al., 2021), microRNAs (Yuan et al., 2015), and signaling molecules (such as MAPKs) (Jia et al., 2016) related to osmotic potential, ion transport, oxidative stress, and other growth regulatory pathways. Although most known transcription factor genes exist in promoters, few studies have specifically analyzed the mechanism by which the entire promoter regulates related genes to protect plants from salt stress. A promoter is likely to contain multiple transcription factor-binding sites and may also contain other gene sequences, including cis-acting elements, that can facilitate the expression and regulation of salt-tolerance genes in plants. Moreover, the cooperation of various favorable genes for salt tolerance in the promoter may improve overall salt tolerance in plants.

In this study, we analyzed the salt-tolerance mechanism of the mangrove tree species *Avicennia marina* (Forsk.) Vierh. via its endogenous promoters. To our knowledge, this represents a novel perspective for studying salt-tolerance mechanisms in plants and the first report on endogenous promoters in mangroves. Our findings can be extended to determine and develop salt-tolerance mechanisms in other plants, potentially enabling the development of new promoter tools and methods for transgenic engineering.

## 2 Materials and methods

### 2.1 Plant materials

Leaves of *A. marina* were collected from the Dongzhaigang National Nature Reserve, Hainan, China. *Arabidopsis thaliana* ecotype Col-0 plants (Kunkel et al., 1993) were cultivated to the flowering stage in a growth chamber (23 °C) with a photoperiod of 18/6 h (light/dark). *Nicotiana tabacum* var. Samsun NN, *Pachyrhizus erosus* (L.) Urb., *Solanum tuberosum* L., *Cocos nucifera* L., *Agropyron cristatum* (L.) Gaertn., and *Thalassia hemprichii* (Ehrenb.) Asch. were all cultured in a growth chamber (26°C) with a photoperiod of 16/8 h (light/dark).

### 2.2 Promoter prediction

The genome sequence of *A. marina* (National Center for Biotechnology Information, GCA_013168755.1) was analyzed using PromPredict to identify promoters (Morey et al., 2011; Bansal et al., 2014; Yella et al., 2018; Das and Bansal, 2019). Many salt-inducible promoters have been predicted, most of which contain the salt-induced cis-acting element GT1-motif (GGTTAA) or dehydration response element DRE (TACCGACAT) (Manavella et al., 2008). Among the endogenous promoters predicted by PromPredict for the whole genome of *A. marina*, we screened out salt-tolerance-related endogenous promoters containing the promoter motifs (TATA-box and CAAT-box) and GT1-motif/DRE.

### 2.3 Verification of AMGT1P33 promoter function

Based on the prediction and screening results of the endogenous promoters of *A. marina*, one putative promoter was randomly selected and named AMGT1P33. AMGT1P33 was amplified from the genomic DNA of *A. marina*. Primers (AMGT1P33F/AMGT1P33R) were designed using Primer Premier 5.0 (Premier Biosoft International, CA, USA) and *Hind*III and *Nco*I restriction sites, and their protective bases were introduced in the primers. The pCAMBIA1304 vector was ligated to AMGT1P33 after digesting the 35S promoter. Then, the recombinant vector pCAMBIA1304-AMGT1P33 was constructed, with AMGT1P33 replacing the 35S promoter for *β*-glucuronidase (*GUS*) gene regulation in pCAMBIA1304. Subsequently, pCAMBIA1304-AMGT1P33 was transformed into *Agrobacterium tumefaciens* strain LBA4404 using triparental hybridization (Wang and Fang, 2002). Finally, transient expression was performed in *N. tabacum* using the *A. tumefaciens* -mediated leaf disk method (Horsch et al., 1985). Expression of the *GUS* gene was observed through GUS staining (Jefferson et al., 1987) to verify the promoter function of AMGT1P33.

### 2.4 Expression range regulated by AMGT1P33

To analyze the expression range of AMGT1P33 regulation, the transient expression of *GUS* was regulated by AMGT1P33 in *P. erosus* tuberous roots, *S. tuberosum* tubers (dicotyledons), *C. nucifera* embryos, and *A. cristatum* and *T. hemprichii* leaf discs (monocotyledons) using *A. tumefaciens*-mediated transformation.

### 2.5 Validation of relative gene transient expression using quantitative real-time-PCR

To investigate the ability of AMGT1P33 to regulate the expression of exogenous genes, RNA was extracted from tobacco leaf discs with transient expression and reverse transcription was performed. cDNA was used as a template, and real-time fluorescence quantitative PCR (qRT-PCR) was performed to verify the transient expression level of the *GUS* gene.

qRT-PCR was performed with a TOptical 96 Real-Time PCR System (Analytik Jena, Jena Germany) according to the following procedure: Step 1, 95 °C for 3 min; Step 2, 39 cycles of 95°C for 10 s, followed by 60°C for 30 s and 72°C for 30 s; Step 3, 72°C for 10 min. 18S ribosomal RNA was used to normalize mRNA levels. Primers 18S-F (5′-CAACCATAACTAGCGA-3′) and 18S-R (5′-AGCCTTTGGACCATCTCC-3′) were designed using Beacon Designer 7. The *GUS* primers used in this study were *GUS*-F (5′-ATCGTGTGGATGAAACTG-3′) and *GUS*-R (5′-TCCGTGTGTGTGTATCGGT-3′) (Chang et al., 2017). Each reaction was set to three repetitions. The relative expression level of *GUS* gene was calculated using the 2^−ΔΔCt^ method (Adnan et al., 2011; Gao et al., 2023).

### 2.6 Generation of pCAMBIA1304-AMGT1P33-*AmBADH* transgenic plants

The salt-tolerance gene *BADH* (1509 bp), which has been reported in *A. marina* (Zhou et al., 2008), was cloned from the genomic DNA of *A. marina*. The *AmBADH* gene was integrated into the recombinant vectors pCAMBIA1304 and pCAMBIA1304-AMGT1P33, and new recombinant vectors pCAMBIA1304-*AmBADH* and pCAMBIA1304-AMGT1P33-*AmBADH* were constructed. The recombinant vectors pCAMBIA1304-*AmBADH* and pCAMBIA1304-AMGT1P33-*AmBADH* were transformed into Arabidopsis through the *A. tumefaciens*-mediated floral-dip transformation method (Dutta et al., 2023). Stable transgenic Arabidopsis were generated. Finally, to verify the success of transformation, we extracted DNA from the leaves of T3 transgenic plants (40 days old) and performed AMGT1P33 PCR, extracted RNA, and performed *AmBADH* and hygromycin B resistance gene RT-PCR.

### 2.7 Histochemical staining for GUS activity in transgenic T3 generation *A. thaliana*

Seeds from the T2 generation of pCAMBIA1304-AMGT1P33-*AmBADH* transgenic *A. thaliana* were germinated to detect stability and eliminate plasmid contamination. Two weeks after sowing, transgenic tobacco seedlings of the T3 generation were obtained, and GUS expression across all organs and tissues was assessed. The formula for the GUS staining solution was a slightly improved version of that employed previously (Jefferson et al., 1987). After staining at 37°C overnight, decolorization was performed three times with 75% ethanol solution to remove chlorophyll before taking images.

### 2.8 Detection of salt tolerance in pCAMBIA1304-AMGT1P33-*AmBADH* transgenic *A. thaliana*

T3 generation pCAMBIA1304-AMGT1P33-*AmBADH* transgenic Arabidopsis plants were screened and identified using hygromycin (150 mg/L) for subsequent experimental analysis. Seeds of pCAMBIA1304-AMGT1P33-*AmBADH* and pCAMBIA1304-*AmBADH* wild-type transgenic Arabidopsis plants were sterilized and cultured in 1/2 MS medium containing 100 mM NaCl (Yao et al., 2016; Ni et al., 2021). Thirty seeds were used for each treatment, and root length was measured after eight days of cultivation at 23°C using ImageJ software. Subsequently, these seedlings were cultivated in soil and irrigated with 100 mM NaCl solution every day. After 20 days, the plant height was measured and the plants were photographed.

### 2.9 Yeast transformation and growth assay

We used the previously designed primer pair (AMGT1P33F/AMGT1P33R) and introduced *Age*I and *Hind*III enzyme restriction sites and their protective bases to replace *Hind*III and *Nco*I. The AMGT1P33 promoter was then amplified and recovered from the genomic DNA of *A. marina* (primers: AMGT1P33YF/AMGT1P33YR). The vector pYES2 (Coolaber, VT018) was double digested with *Age*I and *Hind*III and recovered. The two were linked by T4 ligase and transformed by *Escherichia coli* DH5α competent cells (Transgen, CD201-02), and the recombinant vector pYES2-AMGT1P33 was constructed. The pYES2-AMGT1P33 plasmid was subsequently extracted and double-digested with *Hind*III and *Bam*HI.

Using the reported primers for the *A. marina* salt-tolerant gene *AmBADH* (Zhou et al., 2008), we newly introduced the *Hind*III and *Bam*HI restriction sites with their protective bases, which allowed us to amplify the *AmBADH* gene from cDNA (primers: AmBADHYF/AmBADHYR). The two were linked by T4 ligase and transformed by *E. coli* DH5α competent cells, and the recombinant vector pYES2-AMGT1P33-*AmBADH* was constructed.

Both recombinant vectors, pYES2-AMGT1P33-*AmBADH* and pYES2-AMGT1P33, were transformed into competent cells of the *Saccharomyces cerevisiae* W303a strain and then inoculated onto SD/-Ura selective agar plates containing 0.9 M and 1.5 M NaCl (Zhang et al., 2021b; Zheng et al., 2021) to assess salt tolerance. Recombinant W303a cells containing the pYES2 vector served as a negative control.

### 2.10 Statistical analysis

The average value and standard error of the mean of independent replicates for each treatment was calculated using SPSS version 17.0 software (SPSS Inc., Chicago, IL, USA). All quantitative data were analyzed by Duncan’s multiple range test at a significance level of *P* ≤ 0.01. All figures were drawn using GraphPad Prism 8.

## 3 Results

### 3.1 Endogenous promoter prediction

**Figure 1:**
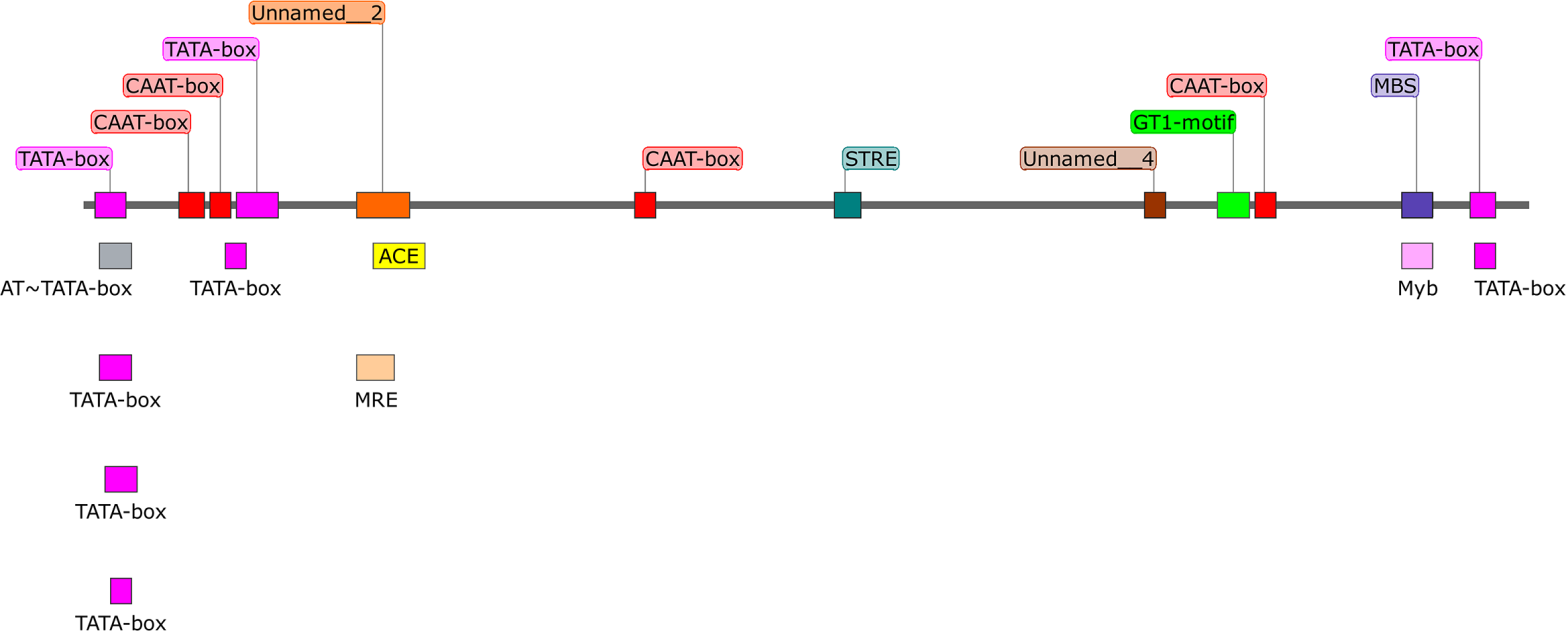
Sequence analysis of the putative promoter region of AMGT1P33 and distribution of cis-acting elements identified in AMGT1P33.

Using PromPredict software, we identified 8,275 suspected endogenous promoters containing the GT1-motif, one of which we named AMGT1P33 (**Table 1**). According to analysis of the AMGT1P33 sequence using PlantCARE, the sequence contained eight TATA-boxes, four CAAT-boxes, and one GT1-motif (**Figure 1**; **Table 2**). No homologous sequences aligning with AMGT1P33 were found in the National Center for Biotechnology Information database or eukaryotic promoter database, confirming AMGT1P33 as a new promoter, which has been assigned GenBank accession number OR677823.

**Table 1:** Results of AMGT1P33 predicted by PromPredict for *A. marina* genome.

Feature	Predicted results
Start of the 1000nt long genomic window position	31292250
%GC of 1000nt long fragment	40.50
pstart	31292317
pend	31292591
length of the promoter	275
lsp	31292476
lspe	-12.26
Dmax pos	31292363
Dmax	2.28
Dave	1.76

**Table 2:** Sequence analysis of the putative promoter AMGT1P33 using PlantCARE.

Cis-acting regulatory elements	Organism^a^	Position^b^	Strand^c^	Sequence	Function
TATA-box	*Brassica napus*	2	+	ATATAT	Core promoter element around -30 of transcription start
	*Arabidopsis thaliana*	27	+	TATA	Core promoter element around -30 of transcription start
	*Brassica oleracea*	4	+	ATATAA	Core promoter element around -30 of transcription start
	*Arabidopsis thaliana*	264	–	TATAA	Core promoter element around -30 of transcription start
	*Arabidopsis thaliana*	3	+	TATATA	Core promoter element around -30 of transcription start
	*Oryza sativa*	29	+	TACATAAA	Core promoter element around -30 of transcription start
	*Arabidopsis thaliana*	5	+	TATA	Core promoter element around -30 of transcription start
	*Arabidopsis thaliana*	265	–	TATA	Core promoter element around -30 of transcription start
CAAT-box	*Pisum sativum*	18	+	CAAAT	Common cis-acting element in promoter and enhancer regions
	*Nicotiana glutinosa*	105	+	CAAT	Common cis-acting element in promoter and enhancer regions
	*Nicotiana glutinosa*	24	+	CAAT	Common cis-acting element in promoter and enhancer regions
	*Nicotiana glutinosa*	223	+	CAAT	Common cis-acting element in promoter and enhancer regions
GT1-motif	*Arabidopsis thaliana*	216	+	GGTTAA	light responsive element
ACE	*Petroselinum crispum*	55	+	CTAACGTATT	cis-acting element involved in light responsiveness
AT~TATA-box	*Arabidopsis thaliana*	3	+	TATATA	
MBS	*Arabidopsis thaliana*	251	–	CAACTG	MYB binding site involved in drought-inducibility
MRE	*Petroselinum crispum*	52	+	AACCTAA	MYB binding site involved in light responsiveness
Myb	*Arabidopsis thaliana*	251	–	CAACTG	
STRE	*Arabidopsis thaliana*	143	–	AGGGG	
Unnamed_2	*Petroselinum hortense*	52	+	AACCTAACCT	
Unnamed_4	*Petroselinum hortense*	202	–	CTCC	

a Organism: organisms with this cis-acting element that have been reported;

b Position: the position of this cis-acting element in AMGT1P33;

c Strand: the presence (+) or absence (-) of this cis-acting element exists in the sense or antisense strand of the *A. marina* genome.

### 3.2 Verification of AMGT1P33 promoter function

**Figure 2:**
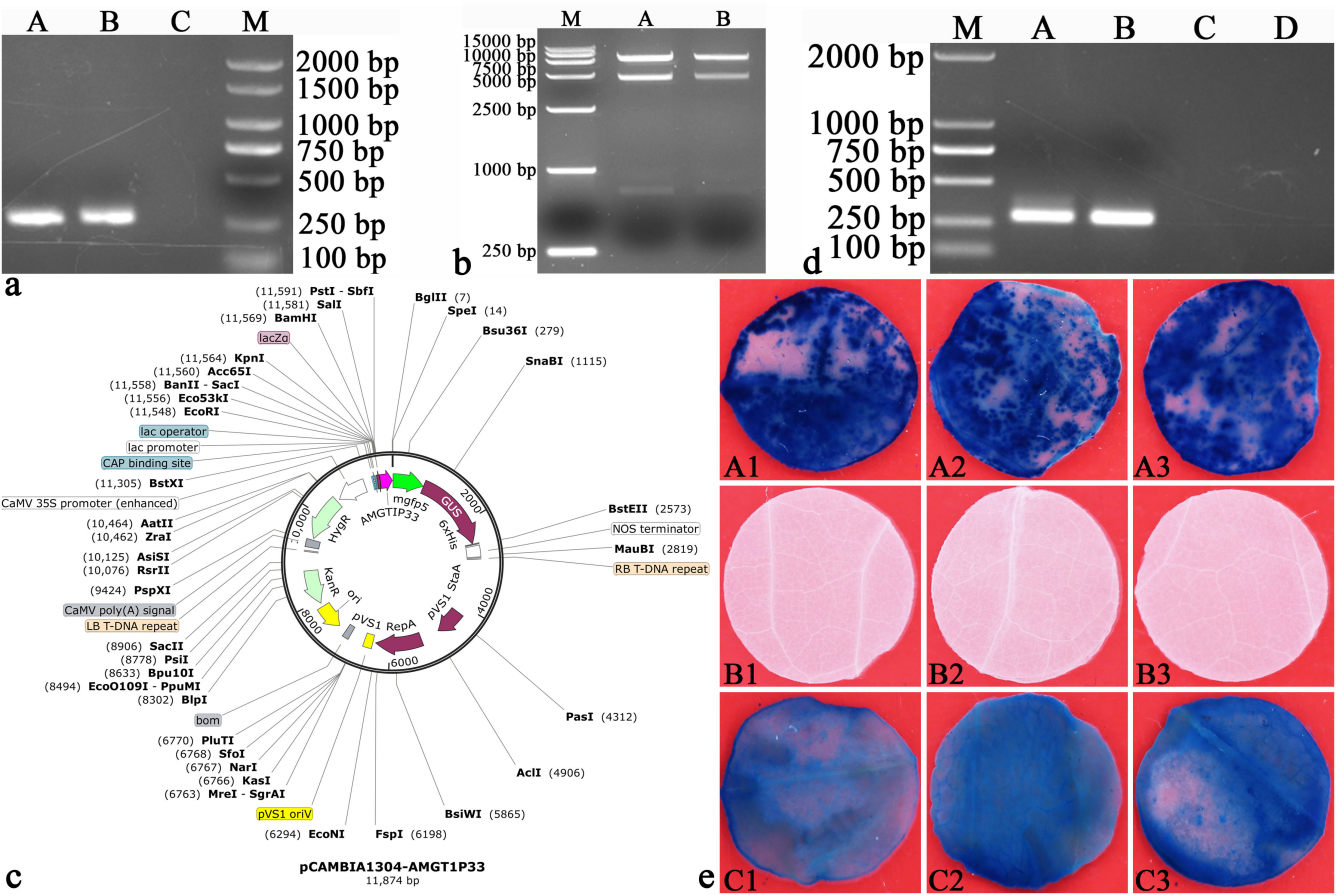
Verification of AMGT1P33 promoter function. **(A)** Amplification of AMGT1P33. AMGT1P33 (275 bp) was amplified from the genome of *A marina* by primers AMGT1P33F/AMGT1P33R, which were introduced with *Hind*III and *Nco*I restriction sites and their protective bases. **(A, B)** AMGT1P33; **(C)** CK^–^, negative control with ddH_2_O as template; (M) Marker 2000. **(B)** Double digestion (*Hind*III and *Nco*I) of pCAMBIA1304 vector (12361 bp). (M) Marker 15000; **(A, B)** pCAMBIA1304 vector. **(C)** Map of recombinant plant expression vector pCAMBIA1304-AMGT1P33. AMGT1P33 was used to replace the 35S promoter, in order to regulate the expression of the GUS gene. **(D)** Colony PCR detection of endogenous suspected promoter TA clone of *A marina*. M: Marker 2000; **(A, B)** endogenous suspected promoter of *A marina* (AMGT1P33, 275 bp); **(C, D)** CK^–^, negative control with ddH_2_O as template. **(E)** Histochemical staining for GUS activity in transiently transformed leaf discs of *N. tabacum*. (A1)–(A3) GUS activity in leaf discs regulated by the AMGT1P33 promoter; (B1)–(B3) CK^–^, negative control, leaf discs of *N. tabacum* wild type; (C1)–(C3) CK^+^, positive control, GUS activity in leaf discs regulated by the 35S promoter.

The electrophoresis of amplified AMGT1P33 using primer AMGT1P33F/R (5’-CCCAAGCTTACATATATAACTTTTTACCAA-3’/5’-CATGCCATGGATTTAATATAAGGTTGTCCAACTGT-3’) showed a single clear PCR product of 275 bp (**Figure 2A**). The electrophoresis results of pCAMBIA1304 vector digestion are shown in **Figure 2B**. The AMGT1P33 sequence was successfully introduced into the pCAMBIA1304 vector, and the plant expression vector pCAMBIA1304-AMGT1P33 was constructed (**Figure 2C**). The results of colony PCR detection after TA cloning are shown in **Figure 2D**.

Regarding the transient expression of AMGT1P33 in tobacco, the tobacco leaf discs with transiently expressed pCAMBIA1304-AMGT1P33-*AmBADH* turned blue after GUS staining. This indicates that the *GUS* reporter gene regulated by AMGT1P33 was successfully expressed, producing GUS, which decomposes 5-Bromo-4-chloro-3-indolyl-beta-D-glucoside (X-Gluc) to form a blue substance. As a negative control for *GUS* expression, the wild-type leaves of *N. tabacum* were not stained blue. As a positive control, *N. tabacum* leaf discs expressing the GUS-regulated 35S promoter were also stained blue (**Figure 2E**). These results indicate that the AMGT1P33 sequence functions as an endogenous promoter in *A. marina*. In addition, the staining of AMGT1P33-GUS was significantly stronger than that of 35S-GUS (deeper blue), indicating that the ability of AMGT1P33 to regulate *GUS* expression is much higher than that of 35S.

### 3.3 Exogenous genes can be regulated by AMGT1P33 in monocotyledons and dicotyledons

**Figure 3:**
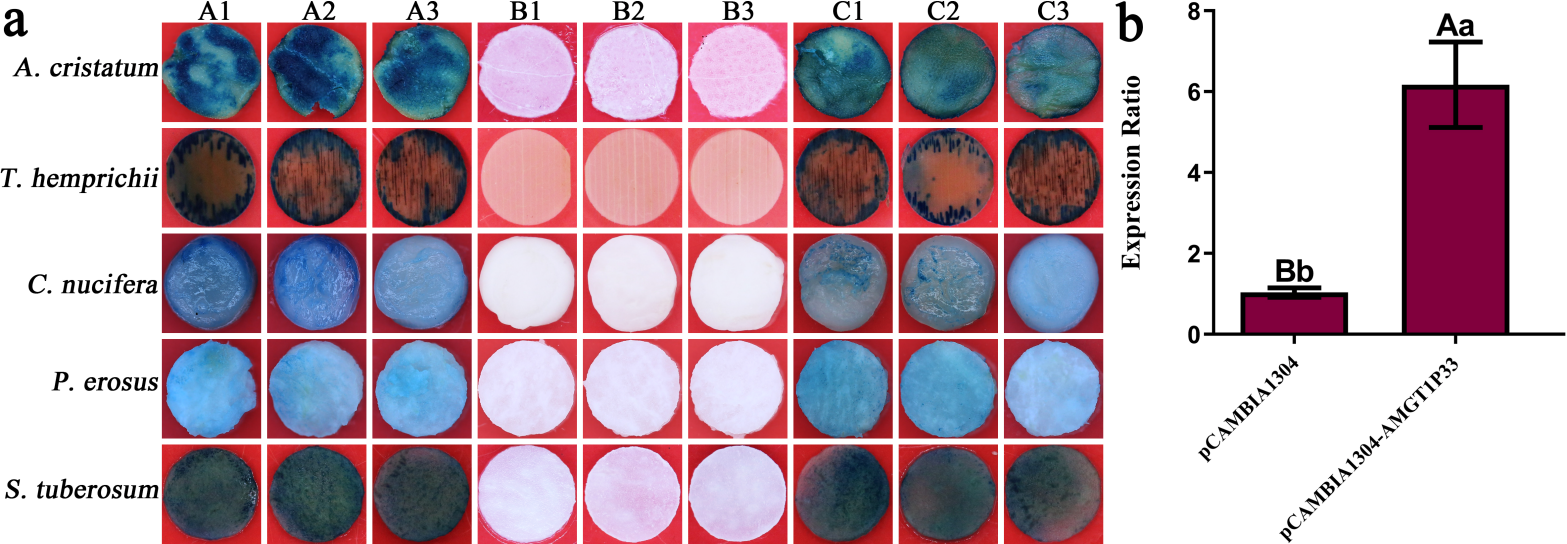
*GUS* transient expression regulated by AMGT1P33 promoter in monocotyledons and dicotyledons. **(A)** Histochemical staining for GUS activity in transient transformed dicotyledons and monocotyledons. (A1) to (A3) GUS activity regulated by the AMGT1P33 promoter; (B1) to (B3) CK^–^, negative control, GUS activity in wild type; (C1) to (C3) CK^+^, positive control, GUS activity regulated by the 35S promoter. **(B)** Transient expression level of *GUS* regulated by AMGT1P33 promoter in *N. tabacum*. Different lowercase letters indicate a significant difference (*P <*0.05), and different uppercase letters indicate a very significant difference (*P <*0.01).

The range of AMGT1P33 regulation by exogenous genes was also examined. Histochemical GUS staining revealed that AMGT1P33 drives the efficient transient expression of exogenous genes (*GUS*) in dicotyledonous plants (*N. tabacum*, *P. erosus*, and *S. tuberosum*) and monocotyledonous plants (*A. cristatum*, *C. nucifera*, and *T. hemprichii*), producing GUS, which decomposes X-Gluc to form a blue-colored substance (**Figure 3A**).

### 3.4 Transient expression regulated by AMGT1P33

The ability of AMGT1P33 to regulate exogenous gene expression was evaluated in greater detail. The RNA of tobacco leaf discs was extracted after transient expression. qRT-PCR was performed using the cDNA obtained through reverse transcription of the RNA as a template to analyze the expression of *GUS* regulated by AMGT1P33 in tobacco. According to the results, the expression of *GUS* in tobacco regulated by AMGT1P33 was 5.97 times higher than that regulated by 35S (**Figure 3B**).

### 3.5 Histochemical staining for GUS activity in transgenic T3 generation *A. thaliana*

**Figure 4:**
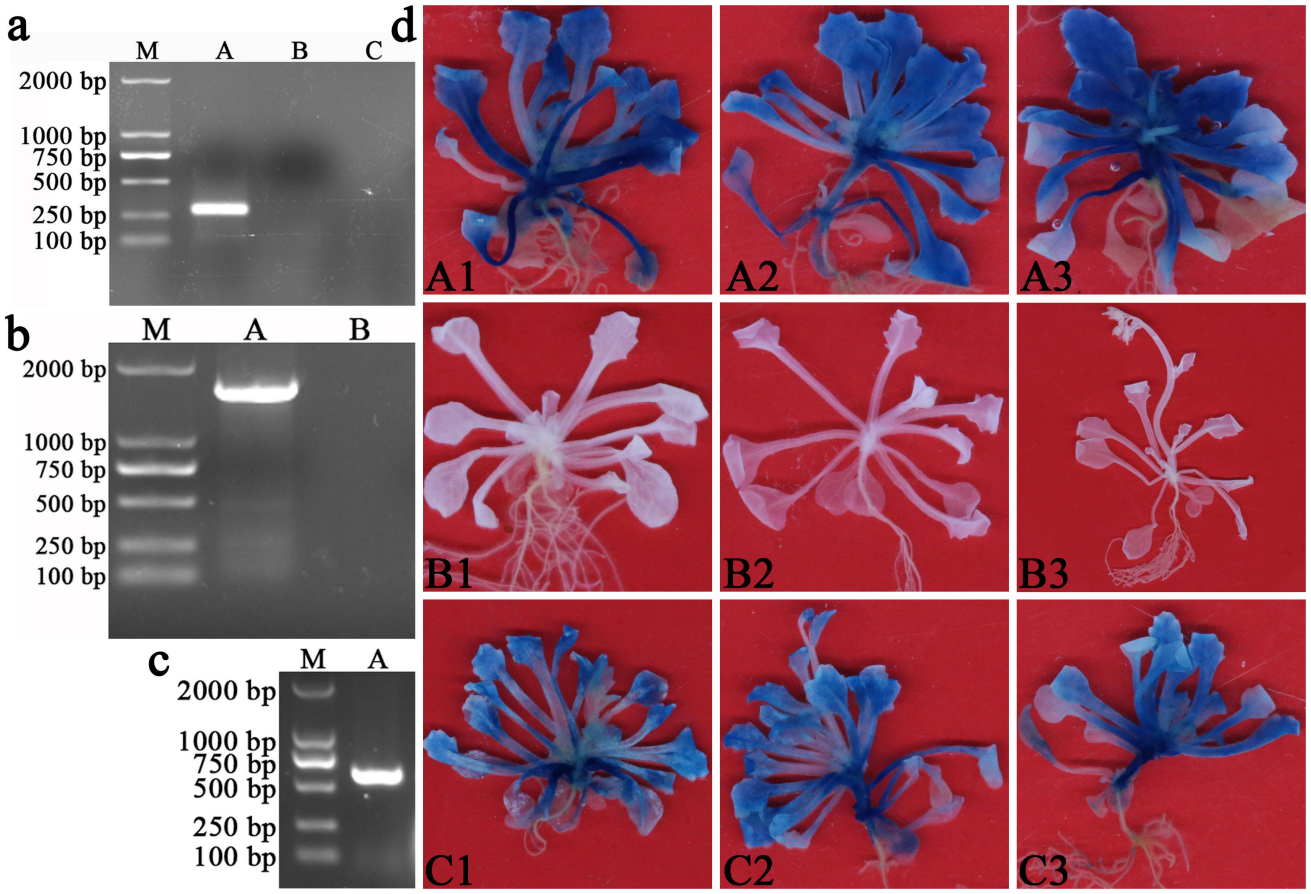
Validation of T3 generation pCAMBIA1304-AMGT1P33-*AmBADH* transgenic *Arabidopsis thaliana*. **(A)** PCR detection of AMGT1P33 in the DNA of T3 generation transgenic Arabidopsis. M: Marker 2000; (A) AMGT1P33 (275 bp); (B) CK^–^, negative control with pCAMBIA1304 transgenic Arabidopsis; (C) CK^–^, negative control with ddH_2_O as template. **(B)** RT-PCR detection of *AmBADH* (1509 bp) in the DNA of T3 generation transgenic Arabidopsis. M: Marker 2000; (A) *AmBADH*; (B) CK^–^, negative control with ddH_2_O as template. **(C)** RT-PCR detection of hygromycin B resistance gene (598 bp) in the DNA of T3 generation transgenic Arabidopsis. M: Marker 2000; (A) hygromycin B resistance gene. **(D)** Histochemical staining for GUS activity in T3 generation pCAMBIA1304-AMGT1P33-*AmBADH* transgenic Arabidopsis. (A1)–(A3) GUS activity in *A. thaliana* regulated by the AMGT1P33 promoter; (B1)–(B3) CK^–^, negative control, *A thaliana* ecotype Col-0 wild type; (C1)–(C3) CK^+^, positive control, GUS activity in *A thaliana* regulated by the 35S promoter.

The PCR results showed that AMGT1P33 had a single clear band in T3 transgenic Arabidopsis DNA (**Figure 4A**). The RT-PCR results showed that both *AmBADH* and the hygromycin B resistance gene had a single clear band in T3 transgenic Arabidopsis DNA (**Figures 4B, C**). Subsequent GUS staining results showed that the reporter gene *GUS* regulated by AMGT1P33 was successfully expressed in Arabidopsis, producing GUS, which decomposes X-Gluc to form a blue substance. As a negative control, *A. thaliana* ecological wild-type Col-0 plants were not stained blue. As a positive control, transgenic Arabidopsis plants regulated by 35S for *GUS* expression (pCAMBIA1304 vector) were also stained blue (**Figure 4D**). These results indicated that AMGT1P33 was successfully transformed into Arabidopsis and that AMGT1P33 effectively drives *GUS* gene expression.

### 3.6 pCAMBIA1304-AMGT1P33-*AmBADH* transgenic *A. thaliana* exhibits stronger tolerance to salt stress than pCAMBIA1304-*AmBADH* transgenic *A. thaliana*

**Figure 5:**
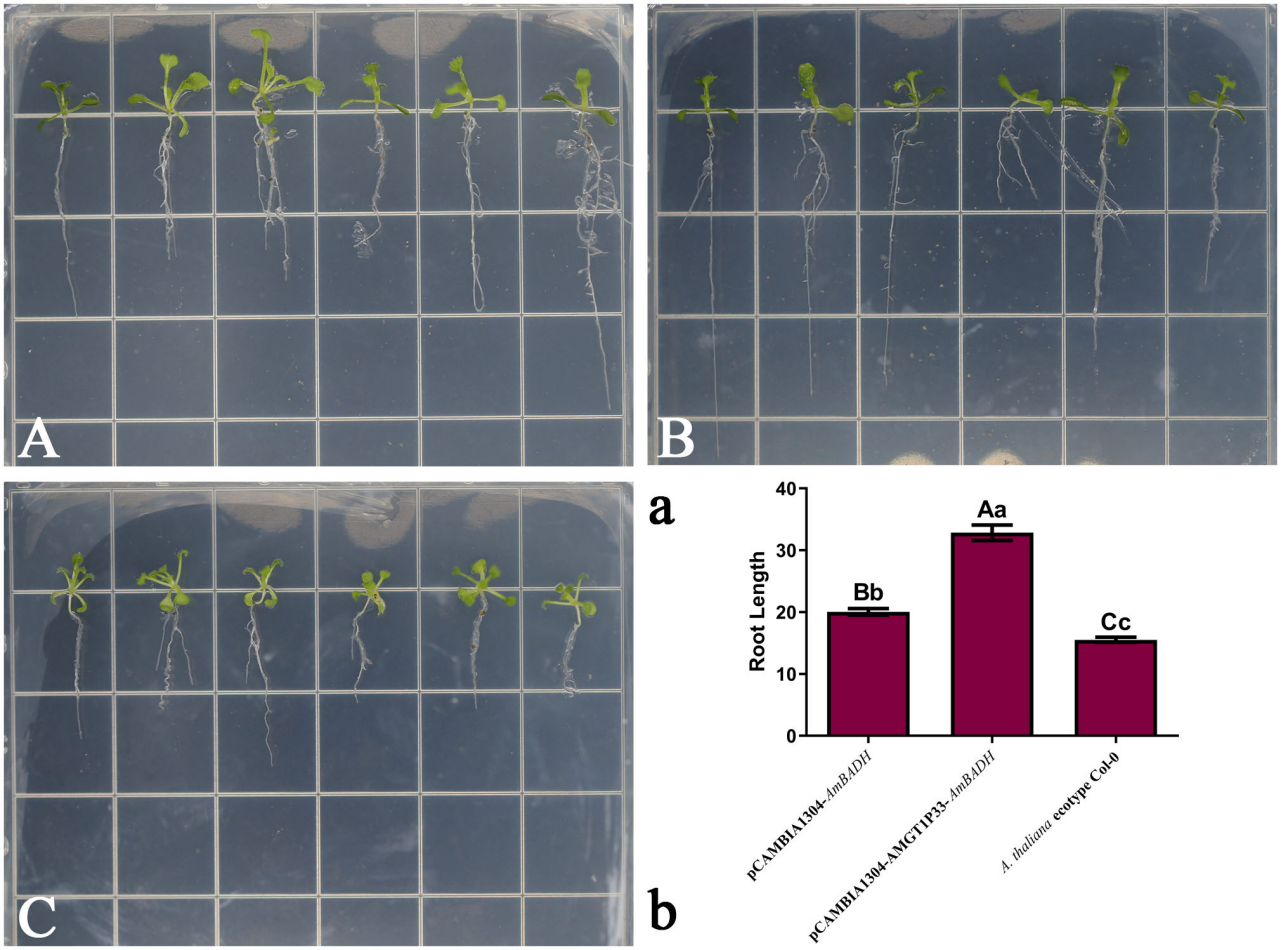
Salt stress analysis of pCAMBIA1304-AMGT1P33-*AmBADH* transgenic plants based on seedling average root length. Ten-day-old seedlings were transplanted into MS medium containing 100 mM NaCl, were then grown for 7 d before measuring the root length. **(A)** Photographs of transgenic lines and *A. thaliana* ecotype Col-0 wild type seedlings on MS medium with NaCl. (A) pCAMBIA1304-*AmBADH* transgenic *A thaliana*; (B) pCAMBIA1304-AMGT1P33-*AmBADH* transgenic *A. thaliana*; (C) *A. thaliana* ecotype Col-0 wild type. **(B)** Seedling average root length (mm) of WT and transgenic lines under NaCl stress after 7 d Different lowercase letters indicate a significant difference (*P <*0.05), and different uppercase letters indicate a highly significant difference (*P <*0.01).

**Figure 6:**
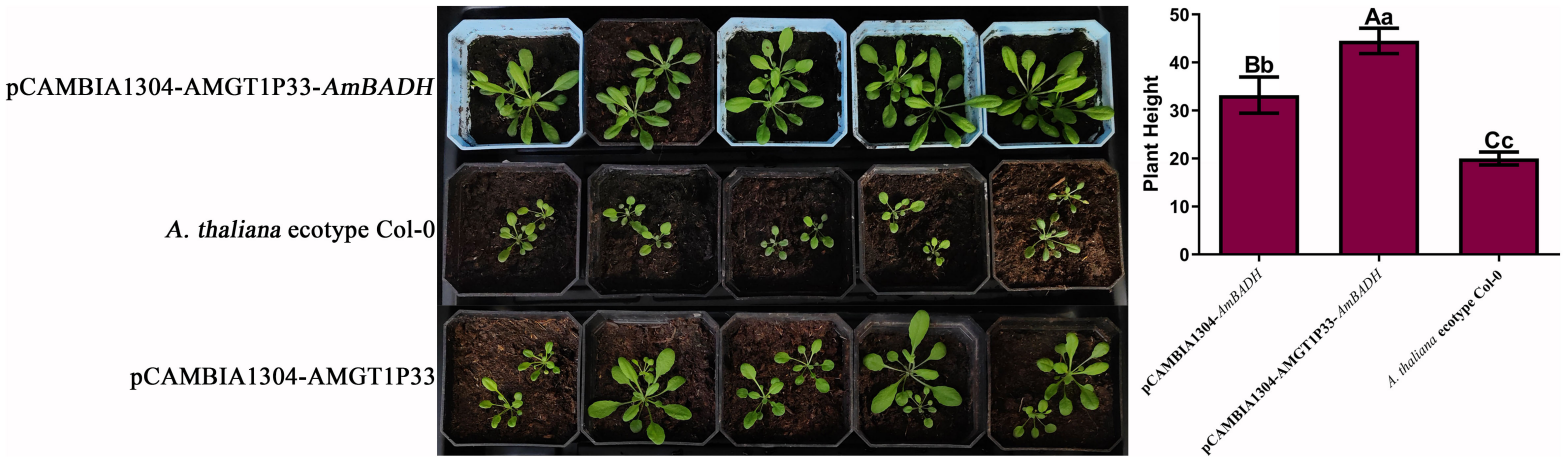
Salt stress analysis of pCAMBIA1304-AMGT1P33-*AmBADH* transgenic plants based on plant height. Eight-day-old seedlings were transplanted into nutrient soil, irrigated with 100 mM NaCl every day, and were then grown for 20 d before measuring the plant height. **(A)** Photographs of transgenic lines and *A. thaliana* ecotype Col-0 wild type plants on soil. (A) pCAMBIA1304-AMGT1P33-*AmBADH* transgenic *A. thaliana*; (B) *A. thaliana* ecotype Col-0 wild type; (C) pCAMBIA1304-*AmBADH* transgenic *A. thaliana*. **(B)** Average plant height (mm) of WT and transgenic lines under NaCl stress after 20 d Different lowercase letters indicate a significant difference (*P <*0.05), and different uppercase letters indicate a very significant difference (*P <*0.01).

Salinity experiments showed that the salt tolerance of both pCAMBIA1304-AMGT1P33-*AmBADH* and pCAMBIA1034-*AmBADH* transgenic Arabidopsis plants was significantly higher than that of Arabidopsis ecological wild-type Col-0 plants. Notably, the salt tolerance of pCAMBIA1304-AMGT1P33-*AmBADH* transgenic Arabidopsis plants was significantly higher than that of pCAMBIA1034-*AmBADH* transgenic Arabidopsis (**Figures 5A**, **6A**). The average root length of the T3 generation seeds of pCAMBIA1304-AMGT1P33-*AmBADH* transgenic Arabidopsis after 8 days of germination was 1.5 times that of the pCAMBIA1304-*AmBADH* transgenic plants; this difference was highly significant (**Figure 5B**). After growing in soil under NaCl stress for 20 days, the plant height of pCAMBIA1304-AMGT1P33-*AmBADH* transgenic Arabidopsis was 1.34 times that of pCAMBIA1304-*AmBADH* transgenic plants; this difference was significant (**Figure 6B**).

### 3.7 pYES22-AMGT1P33-*AmBADH*-transformed yeast exhibits enhanced salt tolerance

**Figure 7:**
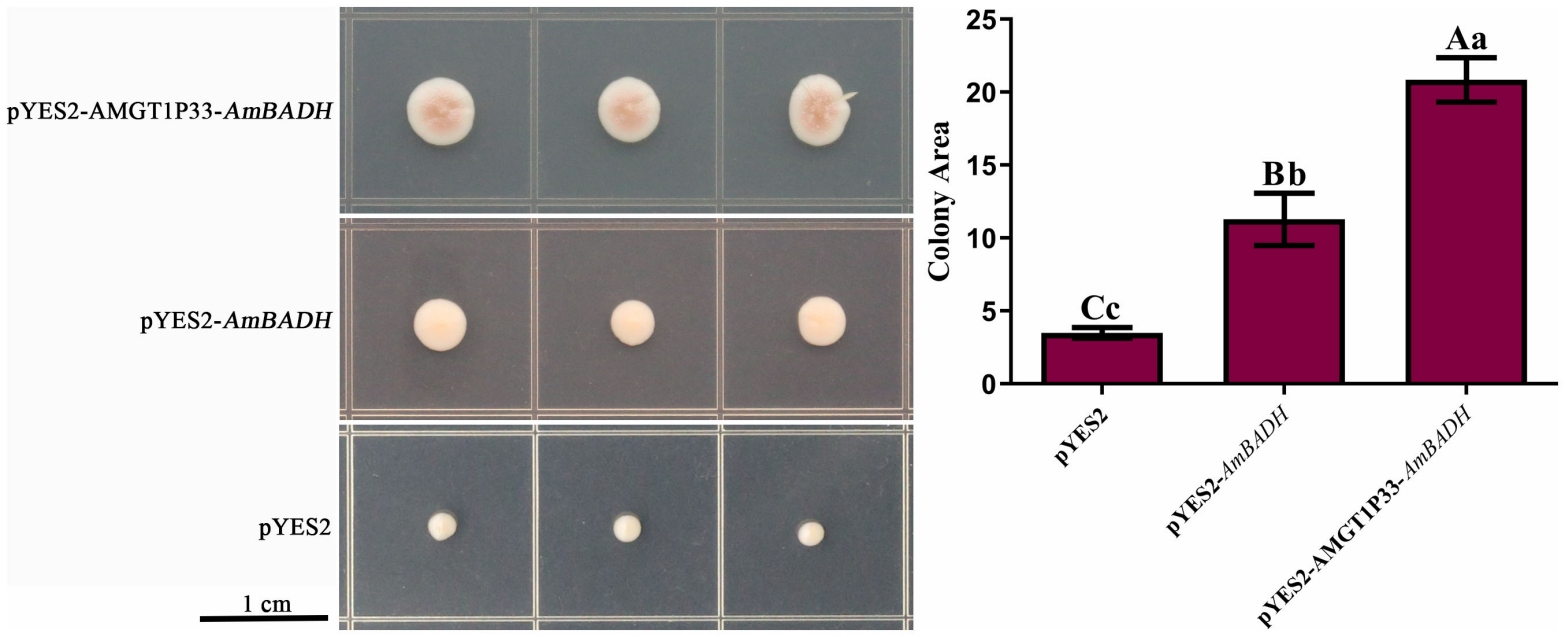
Salt stress analysis of pYES2-AMGT1P33-*AmBADH* transgenic yeast based on colony area. Single colonies of *S. cerevisiae* were inoculated onto SD medium plates containing 1.5 M NaCl, were then grown for 8 d before measuring the colony area. **(A)** Photographs of transgenic strains inoculated on SD medium with NaCl. **(B)** Average colony area (mm^2^) of WT and transgenic strains under NaCl stress after 8 d Different lowercase letters indicate a significant difference (*P <*0.05), and different uppercase letters indicate a very significant difference (*P <*0.01).

The role of AMGT1P33 in the salt-stress response was preliminarily studied using yeast. *S. cerevisiae* strain W303a was transformed with pYES2-AMGT1P33-*AmBADH*, pYES2-*AmBADH*, and the pYES2 empty vector. Although all yeast strains exhibited resistance to low NaCl concentrations, yeast with pYES2-AMGT1P33-*AmBADH* and pYES2-*AmBADH* plasmids grew faster than yeast with empty vectors when the NaCl concentration was increased to 1.5 M. Additionally, yeast with the pYES2-AMGT1P33-*AmBADH* plasmid grew faster than yeast with the pYES2-*AmBADH* plasmid (**Figure 7**). This indicates that the exogenous expression of *AmBADH* can enhance salt tolerance in yeast. Regulation of the expression of the salt-tolerance gene *AmBADH* by the salt-tolerance-related promoter AMGT1P33 led to significantly greater salt tolerance in yeast than regulation by the GAL1 promoter.

## 4 Discussion

Since salt tolerance depends on polygenic traits, controlling this trait through single-gene transformations is challenging (Turan, 2018). Gene combinations can increase salt-stress tolerance (Singla-Pareek et al., 2003; Yang et al., 2009; Ghosh et al., 2014; Pruthvi et al., 2014), where any reaction or biosynthesis of any permeate is catalyzed by a number of enzymatic steps. Therefore, to achieve an efficient response or better production, a polygenic strategy may be more effective for salt tolerance. Similarly, the regulation of ion transport involves multiple protein cascades.

Promoters often contain multiple transcription factor-binding sites and other gene sequences, such as cis-acting elements, that facilitate the expression and regulation of salt-tolerance genes in plants. Moreover, the overall cooperation of various favorable genes for salt tolerance in the promoter may enhance salt tolerance in plants. Therefore, we thoroughly analyzed the salt-tolerance mechanism of *A. marina* through its endogenous promoters. Our results indicated that AMGT1P33 is a highly effective promoter, with a far superior ability to drive exogenous gene expression in tobacco than the 35S promoter (5.97 times). As well as promoting endogenous gene expression in *A. marina*, AMGT1P33 can also facilitate the expression of beneficial exogenous genes. More importantly, AMGT1P33 can be used to express useful exogenous genes in other plants, providing a new tool for genetic engineering. Furthermore, AMGT1P33 has a broad transcriptional regulatory range, driving exogenous *GUS* expression in dicotyledons such as *N. tabacum*, *P. erosus*, and *S. tuberosum*, as well as monocotyledons such as *A. cristatum*, *C. nucifera*, and *T. hemprichii*.

Effective regulation of the stable expression of exogenous salt-tolerance genes in Arabidopsis by the AMGT1P33 promoter is particularly encouraging. The salt tolerance of *AmBADH* transgenic Arabidopsis regulated by the AMGT1P33 promoter was significantly better than that of wild-type Arabidopsis; however, the salt tolerance of AMGT1P33-*AmBADH* transgenic Arabidopsis was significantly higher than that of *AmBADH* transgenic Arabidopsis. This indicates that salt-tolerant genes, as well as salt-tolerant endogenous promoters, play a role in the salt-tolerance mechanism of *A. marina*. As research in this area has progressed, specific (inducible and tissue/organ specific) promoters have become more important because of their excellent ability to regulate gene differential expression in time and space compared to constitutive promoters. Thus, these promoters represent key factors in the regulation of gene expression.

Plants respond to environmental stress by interacting with transcription factors and cis-acting elements. The AMGT1P33 promoter sequence contains the GT1 motif, a cis-acting element widely present in many plant gene promoters. Most reported salt-stress promoters contain GT1 or DRE cis-acting elements (Manavella et al., 2008). GT1 is associated with not only salt stress (Park et al., 2004; Zheng et al., 2012; Duan et al., 2016; Wang et al., 2017; Wang et al., 2023) but also biotic and abiotic stresses such as drought stress, osmotic pressure stress, disease resistance, and light response (Kumar et al., 2009). This indicates that the AMGT1P33 promoter has great research potential. Li et al. identified that OsASR2 was specifically bound to GT-1 in rice and overexpression of *OsASR2* enhanced the resistance against *Xanthomonas oryzae* pv. oryzae and *Rhizoctonia solani*, and tolerance to drought in rice (Li et al., 2018). Park et al. found that an interaction between a GT-1 cis-element and a GT-1-like transcription factor plays a role in pathogen- and salt-induced SCaM-4 gene expression in both soybean and Arabidopsis (Park et al., 2004). In the following research, we can investigate the transcription factors that bind to GT1 of AMGT1P33 in *A. marina*, as well as the effects of their interactions on the drought resistance and disease resistance of *A. marina*, through methods such as yeast mononucleosis, immunoprecipitation, deletion mutations, and overexpression. Further investigate whether the expression of disease resistant or drought resistant genes regulated by AMGT1P33 in crops can improve crop yield and safety.

In summary, this study represents a novel perspective for studying salt-tolerance mechanisms in plants and is, to our knowledge, the first report of endogenous promoters in mangroves. We predicted, screened, and validated an endogenous promoter associated with salt tolerance in *A. marina*. AMGT1P33 effectively drives exogenous gene expression in both monocotyledonous and dicotyledonous plants. In Arabidopsis, the expression of *GUS* regulated by the AMGT1P33 promoter was 5.97 times higher than that regulated by the 35S promoter. Moreover, AMGT1P33-mediated regulation of *AmBADH* significantly improved salt tolerance in yeast and Arabidopsis. Our findings advance our understanding of the salt-tolerance mechanisms in *A. marina* and can provide a reference for elucidating salt-tolerance mechanisms in other plants.

## Data Availability

The datasets supporting the conclusions of this article have been submitted to NCBI Nucleotide database (https://www.ncbi.nlm.nih.gov/nuccore/) under the accession number OR677823.
